# Sedentary images in a popular US based parenting magazine: 2010-2015

**DOI:** 10.15171/hpp.2016.10

**Published:** 2016-06-11

**Authors:** Corey H. Basch, Aleksandar Kecojevic, Valerie Cadorett, Emily A. Zagnit

**Affiliations:** ^1^Department of Public Health, William Paterson University, Wayne, NJ 07470, USA; ^2^Graduate Student, Teachers College, Columbia University, New York, NY, USA

**Keywords:** Sedentary, Physical inactivity, child health, media

## Abstract

**Background: ** Parenting magazines serve as a platform for advertisers to target children and their caregivers. The purpose of this study was to analyze and describe the number of pictures of sedentary and non-sedentary children pictured in the popular parenting magazine, Parents.

**Methods:** Our sample consisted of 72 issues from Parents magazine from January 2010 to December 2015. The sampling frame consisted of all printed issues over this time period. All pictures of children, whether they were in advertisements or models appearing throughout the magazine were included. There were a total of 11 018 children images reviewed.

**Results: ** The overwhelming majority included sedentary children (n = 9734, 88.3%), while the minority showed children engaged in some kind of activity (n = 1284, 11.7%).

**Conclusion:** Parents are encouraged to discuss with their pediatricians which activities are most beneficial for their children’s health.

## Introduction

Obesity is a worldwide health issue, and the World Health Organization (WHO) reports that, across the globe, it has doubled since 1980.^1^ In the United States in last 30 years, childhood obesity doubled, while there was a fourfold increase of obesity among adolescents.^2^ About 1 in 6 American children between the ages of 2-19 years old are obese, with a greater effect on ethnic and racial minority groups.^3^ Children who are overweight between the ages of 2-5 years old were four times more likely to be overweight adults.^4^

Extensive research on the deleterious effects of obesity on a person’s health, both short- and long-term, indicates evidence of heart disease, cancer, and type 2 diabetes.^3^ Obese children and adolescents have a higher risk for joint issues, sleep apnea, and social and psychological problems like poor self-esteem.^2^ They also have a higher chance of having pre-diabetes.^2^ The rise in obesity and weight gain can be attributed to many things, including the cost and access to healthy food and the amount of time spent being active.

Children are an influence market because they have the power to impact family purchasing choices and are a future market because when they grow up they will be adult consumers.^5^ In 2009, the Federal Trade Commission (FTC) stated that 1.8 billion dollars were spent by 48 companies to advertise food and drinks to children in the United States.^6^ In 2006, the FTC noted that 44 food and drink companies spent $2.1 billion, which includes the “cost of fast-food restaurant toys,” to advertise to children as their target audience.^6^

Parenting magazines serve as a platform for advertisers to target children and their caregivers. Magazines, and the advertisements contained within them, serve as a vehicle to convey health information and possibly may influence numerous parents. The purpose of this study was to analyze and describe the number of pictures of sedentary and non-sedentary children pictured in the popular parenting magazine, *Parents.* This study did not involve human subjects, and was viewed as an exempt study by the Institutional Review Board (IRB) at William Paterson University.

## Materials and Methods

### Sample

*Parents* is a widely read parenting magazine with an audience of more than 13 million readers each month.^7^ Sixty-five percent of readers made a direct purchase as a result of reading an ad published in the magazine.^7^ The median reader age is 36 and the female to male reader ratio is 85:15.^7^ A full page 4-color advertisement in a 2016 *Parents* magazine costs close to a quarter million dollars.^8^ Our sample consisted of 72 issues from *Parents* magazine from January 2010 to December 2015. The sampling frame consisted of all printed issues over this period.

### Coding

A coding sheet was adapted from prior studies conducted reviewing advertisements in this magazine.^9^ All pictures of children, whether they were in advertisements or models appearing throughout the magazine were included. Tear-out promotions were excluded. Front and inside covers were not included in the page count. However, the back cover was included in the page count. Coding began by recording the page counts for each magazine. We examined the overall occurrence of pictures of children. We then counted the number of pictures of sedentary children and the number of pictures of active children. Each child pictured was coded to determine if she/he was depicted as sedentary or active in the photograph. Sedentary was defined as not moving, i.e. standing, sitting, laying and laying. Active was defined as moving—jumping, running, skipping, etc. All children appearing to be under the age of 13 were included. Cartoon children, and those in which if half the body or more was not shown were not included in the study.

### Data analysis

Data analysis methods utilized descriptive statistics, including calculations of frequencies, percentages, means, and standard deviations. Frequency of each type of advertisements over multiple years was analyzed using analysis of variance (ANOVA) test. Data were analyzed using the SPSS version 23.0.

## Results

There were a total of 11018 children images reviewed. The overwhelming majority included sedentary children (n=9734, 88.3%), while the minority showed children engaged in some kind of activity (n=1284, 11.7%). Table 1 shows the frequency and percentage of advertisements by year, which followed a general downward trend from 2010 to 2015. However, no significant difference in frequency of either sedentary or active children was noted over six years of observations.

**Table 1:** The frequency and percentage of advertisements depicting children in Parents magazine from 2010-2015

**Year**	**Total number of pictures with children**	**Number of pictures with sedentary children (%)**	**Number of pictures with active children (%)**
2010	1707	1438 (84.2)	269 (15.8)
2011	2061	1841 (89.3)	220 (10.7)
2012	1895	1606 (84.7)	289 (15.3)
2013	1987	1800 (90.6)	187 (9.4)
2014	1853	1706 (92.1)	147 (7.9)
2015	1515	1343 (88.6)	172 (11.4)
Total	11018	9734 (88.3)	1284 (11.7)

## Discussion

To our knowledge, this is the first paper to examine the images in a popular US based magazine for parents. Results indicated that children were often depicted as being sedentary, a contributing factor to obesity. A review of literature has found that children and adolescents who are overweight or obese have an increased likelihood of being overweight or obese as adults.^10^ A contributing factor that is significantly linked to predictive obesity is extended hours of sedentary behaviors.^11^ Most US children do not get the recommended 60 minutes of daily physical activity,^12^ and nearly half of US children exceed the recommended two hours of sedentary behavior, including watching TV or playing video games.^11^

Children and adolescents are heavily influenced by their family, friends, and peers, and model their behaviors, sedentary included, based on exposure.^13^ In turn, parents can be heavily influenced by images that are depicted in magazines they read, TV programs they see, or movies they watch. Parents’ magazines are often the primary places where parents get exposed to advertising and countless images. As parents play a critical role in developing children’s physical activity and sedentary behaviors, supporting and encouraging parents through media advertisements can help to reduce the amount of time children stay sedentary. While most advertising companies design their marketing campaigns by portraying children who are physically active, brilliant, fun, and so on, to encourage parents to buy that specific product.^14^ However, we found that the majority of children pictured in this particular magazine were sedentary.

This study is limited by the cross-sectional design, and the fact that there was a single coder. Despite these limitations, this study fills a gap in literature regarding portrayal of activeness among children in a popular US based parenting magazine. Future studies can examine the extent to which this is a global phenomenon. Parents are encouraged to discuss with their pediatricians which activities are most beneficial for their children’s health.

## Ethical Approval

The human subjects committee at William Paterson University does not review studies that do not involve human subjects, such as this project.

## Competing interests

There is no conflict of interest.
